# How Do Cyclodextrins and Dextrans Affect the Gut Microbiome? Review of Prebiotic Activity

**DOI:** 10.3390/molecules29225316

**Published:** 2024-11-11

**Authors:** Anna Gościniak, Emmanuelle Lainé, Judyta Cielecka-Piontek

**Affiliations:** 1 Department of Pharmacognosy and Biomaterials, Poznan University of Medical Sciences, Rokietnicka 3, 60-806 Poznan, Poland; agosciniak@ump.edu.pl; 2 UMR 454 INRAe-UCA, Microbiology, Digestive Environment and Health (MEDIS), Université Clermont Auvergne, 63000 Clermont-Ferrand, France; emmanuelle.laine@uca.fr

**Keywords:** prebiotics, cyclodextrin, dextran, microbiome

## Abstract

The modulation of the gut microbiome through dietary components has garnered significant attention for its potential health benefits. Prebiotics, non-digestible food ingredients that promote the growth of beneficial gut bacteria, play a crucial role in maintaining gut health, enhancing immune function, and potentially preventing various metabolic and inflammatory disorders. This review explores the prebiotic activity of cyclodextrins and dextrans, focusing on their ability to influence gut microbiota composition and function. Both cyclodextrins and dextrans have demonstrated the capacity to promote the growth of beneficial bacterial populations, while also impacting short-chain fatty acid production, crucial for gut health.

## 1. Introduction

Gut health is fundamental to overall well-being, influencing various aspects of our health, from digestion to immune function and even mental health. An imbalance in the gut microbiota, known as dysbiosis, is increasingly associated with a range of chronic conditions, including inflammatory bowel disease, obesity, and metabolic syndrome [1]. One promising approach to address dysbiosis is the use of prebiotics. Recent data show a marked increase in the consumption of prebiotics, reflecting their growing recognition as essential components for maintaining a healthy gut microbiome [2]. Prebiotics serve as food for beneficial bacteria, promoting their growth and activity, which in turn supports gut health and overall physiological balance [3].

In the pharmaceutical industry, numerous excipients are incorporated into formulations primarily to aid in the delivery and stability of active ingredients. Excipients are considered inert, lacking intrinsic biological activity [4]. However, emerging research suggests that some of these substances may possess bioactive properties, potentially contributing to health benefits beyond their conventional roles [5]. When developing new formulations, it is important to be aware of the additional activity of excipients. Attention should be paid in particular to safety issues. Due to the increasing awareness of the scientific community about the microbiome, it is also worth paying attention to the impact of excipients on the gut microbiome, which according to recent reports has a huge impact on health.

Our review paper addresses the use of two excipients, cyclodextrins and dextrans. They were chosen because of their wide use in the preparation of active compound delivery systems. A comparison of characteristics can be found in Table 1. Dextrans are known for their ability to stabilize proteins and extend the shelf life of pharmaceuticals, and use as a drug delivery system like microspheres, microparticles, nanoparticles, nanodroplets, liposomes, micelles and nanomicelles, hydrogels, films, nanowires, bio-conjugates, medical adhesives, and others [6,7]. Cyclodextrins are usually used for enhancing the solubility and bioavailability of drugs [8,9]. In addition to this, they offer several other advantages. Some active molecules are unstable or sensitive to external factors such as light, heat, or oxidation. Cyclodextrins can protect these substances by forming inclusion complexes, which stabilize the active molecules and extend their shelf life [10]. Many drugs also have an unpleasant taste or odor, which can affect patient compliance, especially in children. Cyclodextrins can encapsulate these substances, effectively masking the unpleasant tastes and odors, making it easier for patients to accept and adhere to their medication [11]. Now that these compounds are being investigated for their prebiotic potential, preliminary studies indicate that these compounds may foster the growth of beneficial gut bacteria, thus contributing to gut health [12]. Searches of the literature (PubMed, EMBASE, and Google Scholar) were carried out using the keywords “cyclodextrin” and “dextran”. Cross-referencing was conducted with terms such as “digestion”, “short-chain fatty acids”, “fermentation”, “intestinal flora”, and “gut bacteria” “gut microbiome”, “prebiotic activity”, and “gut health”. Articles identified through this indexed search were reviewed and manually screened to select relevant studies.

## 2. Prebiotic Substances

**Figure 1:**
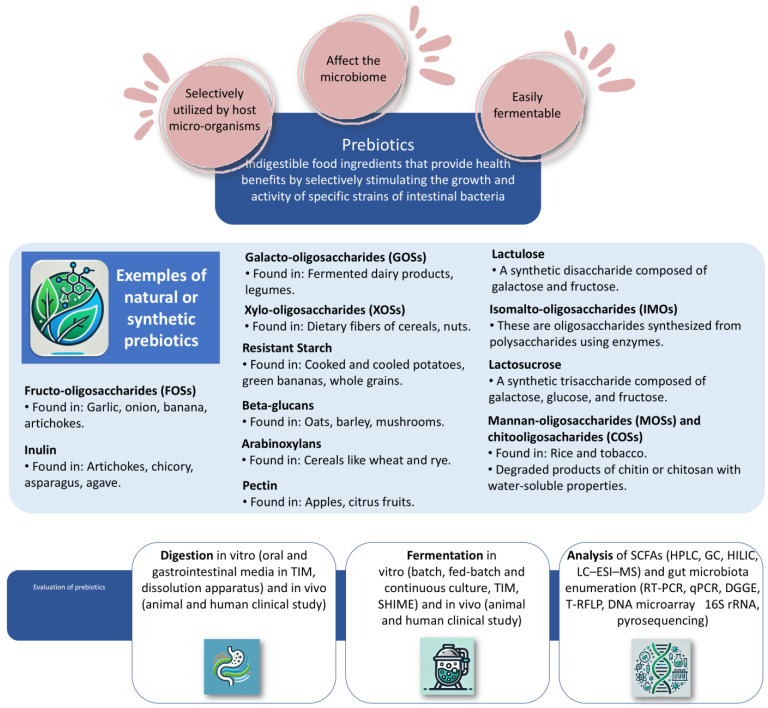
Scheme of prebiotics. TIM: The Netherlands Organization for Applied Scientific Research intestinal model, SHIME: simulator of the human intestinal microbial ecosystem, SCFAs: short-chain fatty acids, HPLC: High-performance liquid chromatography, GC: gas chromatography, HILIC: hydrophilic interaction liquid chromatography, LC–ESI–MS: liquid chromatography–electrospray ionization–mass spectrometry, RT-PCR: real-time polymerase chain reaction, qPCR: quantitative polymerase chain reaction, DGGE: denaturing gradient gel electrophoresis, T-RFLP: terminal restriction fragment length polymorphism, rRNA: ribosomal ribonucleic acid.

Prebiotics are indigestible food ingredients that provide health benefits by selectively stimulating the growth and activity of specific strains of intestinal bacteria, mainly from the *Bifidobacterium* and *Lactobacillus* genera [3]. The term “prebiotic” was first introduced by Gibson and Roberfroid in 1995. They defined prebiotics as “non-digestible food ingredients that beneficially affect the host by selectively stimulating the growth and/or activity of one or a limited number of bacteria in the colon, and thus improve host health.” [28]. Following the initial definition, further research refined the understanding of prebiotics. Scientists focused on identifying specific dietary fibers and non-digestible carbohydrates that met these criteria, such as inulin, fructo-oligosaccharides (FOSs), and galacto-oligosaccharides (GOSs). In 2017, ISAPP (International Scientific Association of Probiotics and Prebiotics) provided a more comprehensive definition: “A prebiotic is a substrate that is selectively utilized by host microorganisms conferring a health benefit.” [29]. This definition emphasizes the selectivity and health benefits conferred by prebiotics and is broad enough to include non-carbohydrate substances and effects beyond the gut microbiota. Today, the understanding of prebiotics includes not only dietary fibers but also other compounds such as polyphenols and fatty acids that can selectively stimulate beneficial microbes [30]. The focus is on the specific health benefits that these compounds can provide to the host, including but not limited to improved digestion, enhanced immune function, and reduced inflammation. A compound is classified as a prebiotic based on the following criteria: it must withstand the acidic conditions of the stomach, remain unaffected by mammalian enzymes, such as amylase, protease, lipase, pancreatic enzymes, and brush border enzymes (e.g., maltase, sucrase, lactase), and not be absorbed in the gastrointestinal tract. Additionally, it should be fermentable by intestinal microbiota and selectively stimulate the growth and/or activity of intestinal bacteria in a way that enhances the host’s health [3]. Examples and properties of prebiotics are shown in Figure 1.

### 2.1. Mechanism of Action

**Figure 2:**
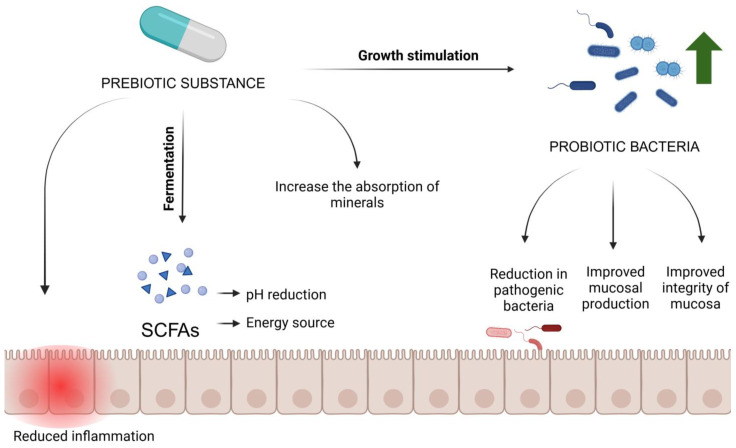
Mechanism of action of prebiotics. SCFAs—Short-chain fatty acids.

Unlike probiotics, which are live micro-organisms, prebiotics are substances that promote the growth of these beneficial bacteria. Their most important feature is that they are indigestible in the upper gastrointestinal tract, allowing them to reach the large intestine unchanged, where they can be fermented by the intestinal microflora [31]. Prebiotics exert their beneficial effects on health through several mechanisms. The main mechanism of action of prebiotics is to provide nutritional substrates for beneficial gut bacteria, especially *Bifidobacteria* and *Lactobacilli*. These bacteria ferment prebiotics, leading to the production of short-chain fatty acids (SCFAs) [32]. SCFAs, such as butyric acid, acetic acid, and propionic acid, are crucial for intestinal health. They act as an energy source for colon epithelial cells and reduce pH, which promotes the growth of beneficial bacteria and inhibits the growth of pathogens. Butyrate also exhibits anti-inflammatory effects by inhibiting the pro-inflammatory cytokines interferon gamma (IFN-γ) and interleukin 2 (IL-2) through inhibition of nuclear factor kappa B (NF-κB) [33]. Probiotic bacteria in the gut, whose growth is stimulated by probiotic or/and prebiotic supplementation, or an appropriate diet, can support intestinal barrier function by stimulating mucus production and contributing to the integrity of the intestinal mucosa [32]. In addition, they can increase the absorption of minerals such as calcium and magnesium and improve lipid metabolism [34]. Regular intake of prebiotics can also reduce the risk of intestinal infections and inflammation, resulting in an overall improvement in gastrointestinal health [35]. The mechanism of action of prebiotics is graphically presented in Figure 2.

### 2.2. Usage of Prebiotics

In pharmaceutical contexts, prebiotics are increasingly formulated into health products available in pharmacies, particularly those targeting digestive health and immune support. They are commonly found in dietary supplements and specialized formulations for infants and children, aiming to support their developing microbiota and overall health. Notably, prebiotics like FOSs and GOSs are included in various pediatric formulas to mimic the beneficial effects of breast milk on infant gut health [36]. Prebiotics are also being incorporated into therapeutic products for adults, especially those designed to manage gastrointestinal disorders such as irritable bowel syndrome (IBS), inflammatory bowel disease (IBD), and chronic constipation [37]. These formulations often aim to restore a healthy balance of the gut microbiota, which can be disrupted by illness, antibiotic use, or poor diet. Furthermore, research has shown that prebiotics can play a role in enhancing the efficacy of probiotics, leading to the development of synbiotic products that combine both prebiotics and probiotics for synergistic health benefits. This combination is believed to improve the survival and colonization of beneficial bacteria in the gut, thereby enhancing their therapeutic effects. In addition to digestive health, prebiotics are being explored for their potential benefits in other areas. The gut–brain axis, which refers to the bidirectional communication between the gut and the brain, has garnered significant attention, and prebiotics are being studied for their potential to influence mood, anxiety, and cognitive function through their impact on the gut microbiota [38,39]. Overall, the application of prebiotics in pharmaceutical contexts is a rapidly evolving field, with ongoing research aimed at uncovering new health benefits and developing innovative products to support overall well-being.

### 2.3. Most Popular Prebiotics

Among the widely recognized prebiotics are oligosaccharides such as FOSs, GOSs, and xylo-oligosaccharides (XOSs) [40,41,42]. These compounds are known to selectively stimulate the growth and activity of probiotic bacteria, predominantly *Bifidobacteria* and *Lactobacilli*, and their metabolites. Prebiotics may also have a direct effect on the gut by improving the intestinal epithelial barrier. Selective enhancement of probiotic bacteria promotes a balanced gut microbiota, which is crucial for various physiological functions including digestion, immune response modulation, and nutrient absorption. Less recognized prebiotics, such as mannan-oligosaccharides (MOSs) and chito-oligosaccharides (COSs), also contribute to microbial balance by enhancing the growth of beneficial bacteria and have been reported to have immunomodulatory effects [43,44]. Recently, marine oligosaccharides have also increased in importance, such as alginate oligosaccharides (AOSs), carrageenan oligosaccharides (KOSs), and agar oligosaccharides (QOSs) [45,46]. Beyond traditional prebiotics, polyphenols exhibit modulatory effects on gut microbiota composition. Although not strictly classified as prebiotics, polyphenols support microbial diversity and favor the growth of beneficial bacteria such as *Bifidobacteria* and *Lactobacilli* [47,48]. Additionally, resistant starch—homopolysaccharide of glucose, a linear molecule of α-1-4-D-glucan, which is resistant to digestion and certain types of dietary fibers, such as β-glucans, acts as substrate for microbial fermentation in the colon, thereby functioning as a prebiotic [49,50].

## 3. Cyclodextrins

**Figure 3:**
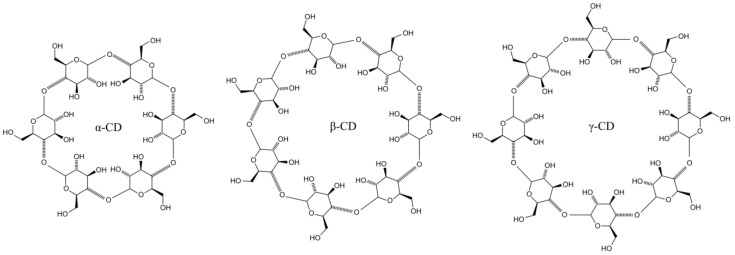
Chemical structure of main types of cyclodextrins.

Cyclodextrins have found widespread use in pharmaceuticals due to their ability to form complexes with a variety of active substances. They are often used in drug formulation to improve the solubility of poorly water-soluble substances, which can lead to an increase in their bioavailability and therapeutic efficacy. In addition, cyclodextrins can be used to mask unpleasant tastes and odors, which can increase the acceptability of drugs to patients [9,51]. The resistance to digestion of cyclodextrins has been studied in various contexts, such as in the presence of prebiotics, where α-cyclodextrins were found to be non-digestible prebiotic carbohydrates supporting beneficial intestinal microflora in the colon. Their digestion depends on the type of cyclodextrin and the presence of specific enzymes in the digestive tract. The structures of main cyclodextrins are presented in Figure 3.

Cyclodextrins α-cyclodextrin and β-cyclodextrin are most likely resistant to digestion by salivary or pancreatic amylase; moreover, they have a fairly low absorption rate in the gut [25]. Therefore, they can enter the large intestine intact and be hydrolyzed by enzymes produced by the intestinal flora. Although the luminal or epithelial enzymes of the gastrointestinal tract are capable of digesting γ-cyclodextrin to glucose, some γ-cyclodextrin or the hydrolyzed fraction is still able to reach the colon [23,24]. The digestibility of cyclodextrins can also affect the stability and release properties of inclusion complexes, impacting the bioavailability of encapsulated compounds.

### 3.1. Impact of Cyclodextrins on Digestion

Studies have shown that α-cyclodextrins also have an effect on patients using such delivery systems, affecting the digestive process. The observed delay in gastric emptying and modification of glycemic and insulin responses suggest that α-cyclodextrin may be an effective tool in managing glucose and insulin levels in the elderly. In the European Union, α-cyclodextrin is recognized as a dietary fiber that reduces post-meal glycemic responses. In the United States, despite being recognized as safe (GRAS), the FDA has not included it in the list of dietary fibers for lack of clear scientific evidence of a beneficial effect on blood glucose levels after a meal [52]. A meta-analysis conducted by Wittowski [52] showed that in the scientific studies reviewed, α-cyclodextrin administration reduces postprandial glycemic responses, but also the lack of increase in insulin levels suggests that α-cyclodextrin acts independently of increasing insulin production and thus the beneficial effect of α-cyclodextrin is not affected by insulin resistance. One of the mechanisms responsible for lowering postprandial glucose concentrations is the inhibitory effect of α-cyclodextrin glucosidase on α-amylase activity [53,54]. α-Cyclodextrin has been shown to bind with dietary fat, potentially aiding in lowering cholesterol levels when taken as a supplement. By binding to bile acids, which play a key role in cholesterol metabolism, α-cyclodextrin promotes the excretion of cholesterol from the body, thereby contributing to reduced cholesterol levels [55].

### 3.2. Prebiotic Activity of Cyclodextrins

α-Cyclodextrin has demonstrated potential as a prebiotic in recent research. Morita et al. [56] found in the clinical trial that α-cyclodextrin enhanced endurance exercise performance in mice and human males. α-cyclodextrin supplementation increased *Bacterioides uniformis* abundance and human male exercise performance. This suggests a potential role for α-cyclodextrin in modulating the gut microbiota to improve physical performance, indicating effects beyond traditional prebiotic functions. In the study conducted by Sakurai et al. [57], apoE-knockout mice, models for atherosclerosis, were fed different diets to observe the effects on atherosclerosis. Analysis of the gut microbiome showed that *Firmicutes* dominated all dietary groups (42.0–63.8%), and *Bacteroidetes* were second in abundance. The *Bacteroidetes*/*Firmicutes* ratio increased in diets with additions of α-cyclodextrin, oligofructose-enriched inulin, and β-cyclodextrin compared to the high-fat Western diet. The higher the ratio of these strains, the greater the health effect [58]. It was observed that *Proteobacteria* were abundantly present when supplemented with β-cyclodextin.

In Nihei et al. [59], male C57BL/6J mice on a high-fat diet with 5.5% α-cyclodextrin gained less weight and fat compared to those on a high-fat diet without α-cyclodextrin. The α-cyclodextrin also increased beneficial gut bacteria such as *Bacteroides*, *Bifidobacterium*, and *Lactobacillus*, that were decreased in the gut microbiota of mice by feeding the high-fat diet. The α-cyclodextrin also increased organic acids and short-chain fatty acids, leading to positive changes in lipid metabolism and anti-obesity effects.

In the study conducted by Guevara et al. [60], α-cyclodextrin supplementation in dogs was investigated for its effects on in vitro fermentation characteristics, nutrient digestibility, fecal microbiota, and serum lipid profiles. Dogs were fed diets with varying amounts of α-cyclodextrin. They found that α-cyclodextrin supplementation did not affect nutrient absorption in the small intestine or fecal microbiota but led to decreased total tract nutrient digestibility and fecal dry matter concentration, indicating altered fermentation in the large intestine which can lead to higher propionate production, as observed in an in vitro experiment by the same authors where evaluation of in vitro fermentation was assessed. The profile of short-chain fatty acid (SCFA) production from cyclodextrin fermentation showed that β-cyclodextrin led to the highest total SCFA and acetate production, γ-cyclodextrin had the highest rate of SCFA and acetate production and the highest butyrate production, and α-cyclodextrin resulted in the highest propionate production but had the lowest SCFA and acetate production and the longest time to reach maximal production rates [60].

A study conducted by Zhu et al. [23] found that dietary cyclodextrin supplementation is an effective strategy for obesity prevention. This dietary pattern showed better results than cellulose supplementation in non-obese mice on a high-fat diet. The three cyclodextrins supplements—α-cyclodextrin, β-cyclodextrin, and γ-cyclodextrin—had varying efficacies in slowing fat accumulation, promoting energy expenditure, increasing SCFA levels, and altering microbiota composition. The microbiota was investigated by 16S rRNA gene sequencing, indicating that a combination of cyclodextrins significantly increased the relative abundance of *Lactobacillus* and *Akkermansia* in the gut and downregulated the relative abundance of *Allobaculum* and *Ruminococcus*. A noteworthy result of the study is the observation that the consumption of different types of cyclodextrins had different effects on the composition of the gut microbiota. It was observed that α-cyclodextrin decreased the relative abundance of Bifidobacterium, while diets containing β-cyclodextrin and γ-cyclodextrin had the opposite effect. In contrast, a high-fat diet containing α-cyclodextrin increased the abundance of *Lactobacillus*, while β-cyclodextrin decreased the relative abundance of *Allobaculum*.

Yamanouchi et al. [61] showed that α-cyclodextrin could be a promising prebiotic for preventing IBD by improving dysbiosis, inducing an anti-inflammatory response through regulatory T cell (pTreg) induction, and promoting butyrate production. This suggests that α-cyclodextrin may positively impact the gut microbiota and help maintain gut health.

In our previous in vitro studies, prebiotic activity of α-cyclodextrin, methylo-β-cyclodextrin, and hydroxypropyl-γ-cyclodextrin was confirmed for 10 prebiotic strains—*Lactobacillus acidophilus*, *Lactobacillus casei*, *Lactobacillus plantarum*, *Lactobacillus brevis*, *Lactobacillus rhamnosus GG*, *Lactobacillus reuteri*, *Pediococcus pentosaceus*, *Lactococcus lactis*, *Lactobacillus fermentum lf*, and *Streptococcus thermophilus* [12].

However, some research does not confirm prebiotic activity of cyclodextrins. In the study conducted by Sasaki et al. [62], the effect of various prebiotics (indigestible dextrin, α-cyclodextrin, and dextran) on human colonic microbiota was investigated using an in vitro model with a daily dosage of 6 g per person. Analysis of bacterial 16S rRNA gene sequences revealed that this prebiotic dosage did not alter microbiota diversity or composition. This result was consistent with a clinical study where 6 g of prebiotics per day did not change fecal microbiota composition. However, the presence of prebiotics reduced pH and increased acetate and propionate production, indicating that even small amounts of prebiotics can activate colonic microbiota metabolism. We can suspect that positive impact of cyclodextrins on the gut microbiome is also indirect. A summary of studies on the prebiotic properties of cyclodextrins can be found in Table 2.

### 3.3. Safety Profile and Toxicological Effects of Cyclodextrins

Regulatory agencies such as the FDA generally recognize cyclodextrins as safe (GRAS) [21]. At high oral doses (over 1000 mg/kg/day), cyclodextrins can lead to reversible side effects in animals, including diarrhea and cecal enlargement. These effects are thought to be adaptive responses to the high intake of indigestible carbohydrates and osmotically active substances, with limited implications for humans [63]. Clinical studies in humans have shown that consuming HP-β-CD at daily doses of 16–24 g over two weeks may result in a higher frequency of loose stools and diarrhea [64]. Consequently, HP-β-CD is regarded as safe for humans at daily doses below 16 g (approximately 270 mg/kg). This is due to its high osmotic activity and partial digestion by gut bacteria. α-cyclodextrin, due to its more effective digestion by the gut flora, may cause more gas formation compared to other cyclodextrins [27]. The recommended daily oral doses for cyclodextrins include up to 6000 mg for α-CD, 500 mg for β-CD, 10,000 mg for γ-CD, and 8000 mg for HP-β-CD in pharmaceutical use [65]. In preclinical studies, the no-observable-effect level for HP-β-CD is 500 mg/kg/day for rats and 1000 mg/kg/day for dogs over a year, while the no-observed-adverse-effect level for SBE-β-CD is 3600 mg/kg/day in both species over three months [66]. Studies in young rats showed that doses of HP-β-CD up to 2000 mg/kg/day were not more toxic than in adults [67]. Limited data in young children suggest that doses up to 200 mg/kg/day of HP-β-CD over two weeks are well tolerated, with less than 1% oral bioavailability [68].

Some cyclodextrins, especially β-cyclodextrin, can accumulate in the kidneys when administered intravenously or at high doses, potentially leading to renal toxicity. This is partly due to the poor solubility of β-cyclodextrin, which can result in precipitation within renal tubules. Doubts regarding the nephrotoxicity associated with the administration of first-generation β-cyclodextrins have led to warnings and restrictions that have made it difficult to study the nephrotoxicity of later-generation cyclodextrins and have prompted the search for modifications that could reduce this toxicity [69]. β-Cyclodextrin is known to cause hemolysis, particularly when administered intravenously or in high concentrations. This is due to its ability to extract cholesterol and phospholipids from cell membranes, leading to cell lysis [64]. For parenteral use, modified versions like hydroxypropyl-β-cyclodextrin are preferred to mitigate nephrotoxicity risks [8,70]. γ-Cyclodextrin is particularly favored for parenteral administration due to its higher solubility and reduced nephrotoxicity [71]. Nephrotoxicity has been observed in animal models with doses five to ten times higher than those used in certain studies [72].

Some individuals may also experience allergic reactions to cyclodextrins, particularly when they are used in topical products in high concentration [73]. This can manifest as skin rashes or hypersensitivity reactions. Cyclodextrins can interfere with lipid absorption and metabolism due to their ability to form complexes with cholesterol and other lipophilic molecules. However, ingested cyclodextrins are unlikely to interfere with the absorption of fat-soluble vitamins or other lipophilic nutrients. This is due to the reversibility of their complex formation, the effective digestion of γ-cyclodextrin in the small intestine, and findings indicating that even poorly digestible forms like α- and β-cyclodextrin do not reduce the bioavailability of vitamins such as A, D, and E [74,75].

## 4. Dextrans

**Figure 4:**
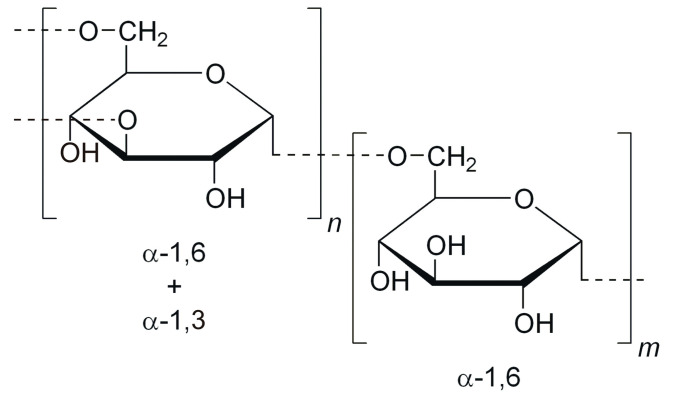
Structure of dextran.

Dextran is a complex branched polysaccharide composed of glucose molecules linked primarily by α-1,6 glycosidic bonds, with branching through α-1,3 glycosidic bonds and, to a lesser extent, α-1,4 and α-1,2 glycosidic bonds [7]. Structure of dextran is shown in Figure 4. Dextran can be categorized based on its branching structure. Linear dextran consists predominantly of α-1,6 linkages, while branched dextran contains α-1,3 linkages, which can influence its digestibility and prebiotic properties [26]. The degree of branching affects the polysaccharide’s resistance to enzymatic hydrolysis and its fermentation by the gut microbiota, making branched dextran particularly interesting for prebiotic applications [76]. Dextran is produced primarily by lactic acid bacteria, particularly, such as *Leuconostoc mesenteroides*, *Weissella cibaria*, and *Streptococcus mutans*, via the enzyme dextransucrase [77]. Dextran’s molecular weight varies widely, from a few thousand to several million Daltons, depending on the source and production method. Its water solubility and biocompatibility make it suitable for various pharmaceutical applications. Two molecular weights of 40 and 70 kDa are most often used in clinical procedures. However, the prebiotic activity of dextran is currently being investigated for different degrees of polymerization of dextran. Dextran is a polysaccharide that is not effectively digested by the human digestive system. The lack of bioavailability after oral administration is due to the large molecular size of dextrans, which prevents them from passing through epithelial junctions, and structure. [26,78]. Dextran’s unique structure contributes to its resistance to enzymatic hydrolysis by human digestive enzymes, particularly amylases, which are primarily adapted to degrade α-1,4 and α-1,6 linkages found in polysaccharides such as starch and glycogen [79]. The structural characteristics of dextran, including its branching and glycosidic linkage types, render it less susceptible to hydrolysis by amylolytic enzymes, which typically target more linear polysaccharides. As a result, dextran passes through the stomach and small intestine largely intact, reaching the colon where it can be fermented by the gut microbiota. Research has shown that dextran’s resistance to enzymatic breakdown in the upper gastrointestinal tract allows it to act as a prebiotic, promoting the growth of beneficial gut bacteria and leading to the production of short-chain fatty acids (SCFAs) during fermentation [7,78].

### 4.1. Use of Dextran

In pharmaceutical chemistry, dextran is widely utilized for its diverse functionalities. Dextran acts as an anticoagulant by reducing blood viscosity and preventing the formation of blood clots. It is sometimes used during surgery to help prevent thromboembolic complications [80]. Dextran is also employed in drug delivery systems to enhance drug solubility, stability, and controlled release profiles [6]. Additionally, it acts as a viscosity modifier in ophthalmic solutions, a cryoprotectant for biological tissues, and a hydrogel former for wound dressings and tissue engineering [20]. Diagnostic applications include dextran-coated nanoparticles that enhance imaging contrast in techniques such as MRI [81]. Products like dextran 40 and dextran 70 are common in medical use, and iron dextran is used to treat iron deficiency anemia [82]. In topical dressings, dextran plays a role in wound healing formulations due to its hydrophilic nature, which helps maintain a moist environment that is essential for wound healing [83]. Regarding its impact on the cutaneous microbiome, dextran could potentially influence the balance of skin microbes by altering moisture levels. As a food additive, dextran acts as a thickener and stabilizer, modifying texture, stabilizing emulsions, and increasing viscosity in products like salad dressings, sauces, and bakery items [84]. In the cosmetics industry, dextran is valued as a humectant, helping to retain moisture in skin-care products such as lotions, serums, and creams, thus improving skin hydration and elasticity. It also serves as a stabilizer in cosmetic formulations, ensuring that ingredients do not separate over time.

### 4.2. Prebiotic Activity

Tingirikari et al. [85] investigated the stimulation of probiotic bacteria growth by dextran, including *Bifidobacterium animalis* subspecies lactis, *Bifidobacterium infantis*, and *Lactobacillus acidophilus*, which was found to be significant and comparable to that induced by commercial inulin. What is more, neither dextran nor inulin enhanced the growth of *Escherichia coli*. The dextran used in their study shows potential as a beneficial prebiotic for health applications.

The study by Lv et al. [86] focused on *Weissella cibaria* RBA12, isolated from pummelo in Northeast India. This bacterium produces a dextran composed of 97% α-(1→6) linkages in the main chain and 3% α-(1→3) branched linkages. Dextran–RBA12 exhibits strong in vitro prebiotic activity by promoting the growth of probiotic Bifidobacterium and *Lactobacillus* species while controlling the growth of non-probiotic enteric bacteria. It shows exceptional resistance to digestive enzymes compared to commercial inulin.

In the study by Amaretti et al. [87], a long linear chain dextran produced by *Weissella cibaria* was compared to inulin regarding their effects on the growth of specific health-related bacterial taxa and the production of organic acids in pH-controlled batch cultures of intestinal microbiota. Quantitative PCR analysis targeting *Lactobacillus*, *Bifidobacterium*, *Prevotella*, *Bacteroides fragilis*, and *Faecalibacterium prausnitzii* revealed varying relative abundances depending on the carbon source, correlating with fermentation product patterns identified by HPLC. Dextran notably increased the relative amounts of *Prevotella* and *Bacteroides*, consistent with a beneficial acetate–propionate ratio, suggesting its potential as a valuable functional ingredient in the food industry.

The study by Olano-Martin et al. [88] found that dextran can support the growth of *Bifidobacteria*, which persisted for up to 48 h. To better mimic the conditions of the large intestine, as a continuation, a study of the fermentation of dextran, oligodextran IV, and maltodextrin was conducted using a three-stage model. This model can characterize proximal, transverse, and distal colons. The use of artificial models of the digestive system in research is very effective, and many such models have been developed to date [89]. They found that oligodextran IV was the most effective substrate, leading to higher levels of *Bifidobacteria* and *Lactobacilli* compared to dextran and maltodextrin.

Another interesting aspect is whether the molecular weight difference has an effect on dextran activity. In the study by Sarbini et al. [76], this aspect has been evaluated. Both 0.5 and 1.0 kDa dextrans exerted similar effects on the gut microbiota. The 0.5 kDa dextran was fermented more rapidly. However, the faster fermentation of the lower molecular weight dextran does not necessarily make it a superior prebiotic compared to the 1.0 kDa variant. The highly branched 1.0 kDa dextran shows promise for sustained activity, particularly in the distal regions of the colon, suggesting its potential for long-term prebiotic efficacy in these areas. In the study by Sip et al. [90], Dextran 40 kDa proved to be the most effective prebiotic for *Bifidobacterium longum*, *Bifidobacterium animalis*, *Faecalibacterium prausnitzii*, *Lactobacillus salivarius*, *Lactiplantibacillus plantarum 299v*, and *Lacticaseibacillus rhamnosus* GG. Dextran 5 kDa exhibited moderate effectiveness for Lactobacillus helveticus, while Dextran 70 kDa showed the lowest impact on bacterial proliferation overall.

Certain probiotic strains, particularly *Lactobacillus*, use dextran to enhance biofilm formation. Biofilms are communities of bacteria that adhere to the mucosal surfaces of the gut, which helps them colonize and persist in the gastrointestinal tract. The presence of dextran encourages these bacteria to form protective biofilms that improve their resistance to environmental stress and improve their adhesion to the gut lining, thus enhancing their probiotic benefits [91]. A summary of studies on the prebiotic properties of dextran can be found in Table 3.

### 4.3. Safety Profile and Toxicological Effects of Dextran

The safety has been well established through extensive clinical use, particularly as a plasma expander, where it has been administered at high doses without significant adverse effects [92,93]. It is biodegradable and safe for the environment [94,95]. In the United States, dextran is classified as Generally Recognized As Safe (GRAS) by the U.S. Food and Drug Administration (FDA) for certain uses in the food industry [96]. This historical context underscores dextran’s suitability for various medical applications, including drug delivery systems and imaging agents. The biocompatibility of dextran is supported by its ability to form stable complexes with various therapeutic agents, which can enhance the efficacy of drug delivery systems while minimizing toxicity. For instance, Jiang and Salem [97] demonstrated that dextran–polyethylenimine conjugates significantly reduced cytotoxicity compared to unmodified polyethyleneimine, highlighting dextran’s potential to mitigate the adverse effects typically associated with polyethyleneimine in drug delivery systems. Similarly, Vittorio et al. [98] noted that dextran is considered non-toxic and inexpensive, making it a favorable candidate for pharmaceutical applications. In their study, catechin and dextran conjugates showed no toxicity. The low toxicity of dextran is further supported by findings from Avazzadeh et al. [99], who reported that magnetite dextran–spermine nanoparticles exhibited less than 9% cytotoxicity even at high concentrations, indicating that dextran can be safely used in nanoparticle formulations. However, some studies have raised concerns regarding the toxicity of dextran in specific contexts. Thuret et al. [100] reported endothelial toxicity associated with dextran, particularly in corneal applications, where prolonged exposure led to adverse effects on corneal endothelial cells. This finding suggests that while dextran is generally safe, its effects can vary based on the specific biological environment and duration of exposure. Moreover, the safety of dextran in drug delivery systems has been emphasized in various studies. Fang et al. [101] highlighted dextran’s role as a biomaterial with low immunogenicity and high hydrophilicity, which enhances its suitability for drug formulations aimed at reducing toxicity and improving pharmacokinetics. Additionally, Bachelder et al. [102] found that acetal-derivatized dextran was non-toxic in preliminary in vitro assays, reinforcing its potential for therapeutic applications. Dextran’s biocompatibility is also evidenced by its long-standing use as a blood volume expander. Studies have shown that dextran formulations are well tolerated in humans, with a low incidence of severe adverse reactions, particularly when compared to other agents [103]. Furthermore, Petrovici et al. [6] emphasized dextran’s non-toxic and biocompatible nature, making it a valuable component in drug delivery systems.

### 4.4. Comparison of Cyclodextrins and Dextran: Toxicological Profiles

Both cyclodextrins and dextran are generally considered safe under appropriate conditions but present specific toxicological concerns, particularly at high doses or in sensitive populations. The following comparison (Table 4) summarizes their key safety concerns.

Cyclodextrins and dextran, both widely used in pharmaceutical and medical applications, exhibit distinct toxicological profiles that warrant careful consideration in clinical and research settings. Cyclodextrins, while generally safe for oral use, pose risks such as hemolysis and nephrotoxicity when used parenterally. The structure and solubility of different cyclodextrin types (e.g., β-CD, α-CD, γ-CD) play critical roles in determining their toxicity, with β-CD being the most nephrotoxic due to its lower solubility. Modifying cyclodextrins, such as hydroxypropylation, helps to reduce these risks, expanding their therapeutic potential. On the other hand, dextran is widely utilized as a plasma volume expander but carries a significant risk of anaphylactic reactions, especially with higher molecular weights. Its use requires pre-treatment strategies to mitigate allergic responses. Additionally, bleeding risks associated with dextran’s antithrombotic effects and renal implications like osmotic nephrosis require close monitoring, particularly in patients with underlying renal or cardiovascular conditions. However, the use of dextran in the context of oral supplementation is a relatively new concept and the safety profile should be monitor.

## 5. Conclusions and Future Perspective

This study analyzed the effects of cyclodextrins and dextrans on the gut microbiome, focusing on their prebiotic potential. Studies have shown that both cyclodextrins and dextrans can modulate the composition of the intestinal microbiota, promoting the growth of beneficial bacteria such as *Bifidobacteria* and *Lactobacilli*. Few studies, however, have addressed how modifications to cyclodextrins may affect their activity. The effectiveness of dextrans, however, depends on their structure, molecular weight, and degree of branching, which affects the rate and extent of fermentation in different regions of the large intestine, as one study has addressed. In addition, current studies are mostly in vitro studies and concrete evidence in clinical trials is still lacking. Clinical trials in humans will be crucial to confirm the results obtained in in vitro and animal models in order to develop effective nutritional strategies using these prebiotics. Substances used for purposes unrelated to gut health may still influence the gut microbiome, potentially offering additional beneficial effects. Given the increasing awareness of the importance of the microbiome, it is crucial to assess how auxiliary substances affect its composition and diversity. Even non-prebiotic substances, such as pharmaceuticals or food additives, can indirectly promote the growth of beneficial gut bacteria. Investigating the impact of these substances may reveal unexpected benefits for metabolic and immune health. It is essential to conduct studies to ensure that substances used in pharmacology or nutrition either support or do not disrupt a healthy microbiome. Understanding these interactions may lead to the development of products that not only fulfill their primary function but also contribute to gut health. Dextran, although widely used in medicine, has not yet been thoroughly studied as a prebiotic substance. However, emerging research indicates that it exhibits prebiotic activity, promoting the growth of beneficial gut bacteria. Given its extensive use in medical applications, understanding its potential effects on the gut microbiome is of growing interest. Future research should focus on exploring this aspect of dextran, especially in the context of its long-term influence on gut health. There are medical conditions in which the use of dextrans can be significantly beneficial, particularly due to their prebiotic properties. Examples of such conditions include IBD and IBS. For individuals suffering from gastrointestinal disorders, dextrans can support gut health by stimulating the growth of beneficial bacteria in the microbiome, which may alleviate symptoms and improve overall gastrointestinal well-being. Currently, dextrans are used as additives in the food industry and can be consciously incorporated into diets, given their positive prebiotic effects. This action can contribute to improved gut health and overall wellness for those with digestive issues. Future research should focus on further understanding the mechanisms by which cyclodextrins and dextrans affect different gut bacterial populations. Assessing the long-term effects of using these compounds as prebiotics, especially in the context of their impact on metabolic and immune health, will also be an important direction. In addition, the question of safety should not be overlooked as, despite the characterization of these substances as safe, the long-term effects on the gut microbiome need to be monitored.

## Figures and Tables

**Table 1:** Comparison of the properties of cyclodextrins and dextran.

Feature	Cyclodextrins (CDs)	Dextran
Chemical Structure	Cyclic oligosaccharides made of 6–8 α-D-glucose units (α-CD, β-CD, γ-CD) in a toroidal shape [13]	Linear polysaccharide primarily composed of α-(1→6)-linked D-glucose with some α-(1→3) branches [7]
Source	Derived from starch via enzymatic treatment (cyclodextrin glycosyltransferase) [14]	Produced by lactic acid bacteria (e.g., *Leuconostoc* spp.) from sucrose [7]
Molecular Weight	Low molecular weight (α-CD~972 Da, β-CD~1135 Da, γ-CD~1297 Da) [15]	High molecular weight (ranging from 3 kDa to over 2000 kDa depending on synthesis)
Solubility	Water-soluble, with a hydrophobic interior and hydrophilic exterior [13]	Water-soluble, highly hydrophilic [7]
Inclusion Complex Formation	Forms inclusion complexes with various hydrophobic guest molecules due to its toroidal shape [16]	Does not form inclusion complexes but can be chemically modified to encapsulate molecules [17,18]
Applications	Drug delivery, solubilization of hydrophobic drugs, molecular encapsulation, food additives, cosmetics [19]	Plasma volume expanders, drug carriers, biodegradable hydrogels, wound dressings, food stabilizers [20]
Toxicity	Generally non-toxic, biocompatible, and FDA-approved for certain uses [21,22]	Biocompatible and biodegradable; used in medical applications (e.g., Dextran 70 in intravenous solutions) [6]
Modification Potential	Can be chemically modified (e.g., hydroxypropyl-β-cyclodextrin, methylated CDs) to improve solubility, stability, or drug release profiles [10]	Can be modified to form hydrogels, nanoparticles, or other delivery systems (e.g., dextran sulfate, oxidized dextran) [6]
Digestion	Resistant to enzymatic degradation in the upper gastrointestinal tract; fermentable by the gut microbiota [23,24,25]	Mostly resistant to enzymatic degradation in the upper gastrointestinal tract; fermentable by the gut microbiota [26]
Pharmaceutical Use	Widely used to improve bioavailability, stability, and solubility of poorly water-soluble drugs [27]	Used as a drug carrier and for its osmotic properties in IV solutions (e.g., Dextran 40, Dextran 70) [20]

**Table 2:** Summary of studies on the prebiotic activity of cyclodextrins.

Model		Dosage	Findings	Authors
Clinical study	36 participants	200 mg/day, α-cyclodextrin 9 weeks	α-cyclodextrin supplementation increased *Bacteroides uniformis* abundance and human male exercise performance	Morita et al. [56]
Preliminary, small scale, open-label clinical study	4 participants	6 g of DEX or α-cyclodextrin 7 days	Supplementation did not significantly affect the microbiota composition in the human colon	Sasaki et al. [62]
Animal model	60 female apoE-KO mice	high-fat diet supplemented with 1.5% (*w*/*w*) a α-cyclodextrin/β-cyclodextrin/oligofructose-enriched inulin 11 weeks	Addition of α-cyclodextrin to the diet of apoE-knockout mice is associated with changes in the gut flora *Bacteroidetes*/Firmicutes ↑ * Clostridium ↓ *Turicibacterium* ↓ *Erysipelotrichi* ↓ *Dehalobacteriaceae* ↑ *Comamonadaceae* ↑ *Peptostreptococcaceae* ↓	Sakurai et al. [57]
Animal model	15 male mice (C57BL/6JJmsSlc)	high-fat diet supplemented with 5.5% (*w*/*w*) α-cyclodextrin 16 weeks	Supplementation of α-cyclodextrin regulates the gut microbiota in high fat diet mice total number of bacteria ↑ *Bacteroides* ↑ *Bifidobacterium* ↑ *Lactobacillus* ↑	Nihei et al. [59]
Animal model	5 female purpose-bred hound-mix dogs	0, 2, 4, 6, and 8 g of α-cyclodextrin/day	Cyclodextrins do not affect dogs’ microbiome	Guevara et al. [60]
Animal model	36 male C57BL/6JNifdc mice	45% high-fat diet that replaced cellulose with γ-cyclodextrin 9 weeks	Significant increase in relative abundance of *Lactobacillus* and *Akkermansia* in the gut and downregulation of the relative abundance of *Allobaculum* and *Ruminococcus*	Zhu et a. [23]
Animal model	C57BL/6J male mice	5% *w*/*v* α-cyclodextrin in drinking water ad libidum 2 weeks	Promotion of the growth of beneficial bacteria such as *Akkermansia muciniphila* and *Bacteroides acidifaciens*, while eliminating pathogenic bacteria such as *Clostridium perfringens*	Yamanouchi et al. [61]
In vitro study	Kobe University Human Intestinal Microbiota Model, KUHIMM	0.2% of indigestible dextrin, α-cyclodextrin, and dextran	Prebiotic additive did not change the diversity and composition of colonic microbiota; their addition reduced the pH and increased the generation of acetate and propionate in the in vitro system	Sasaki et al. [62]
In vitro study	Human fecal samples from 3 individual male volunteers	500 mg of α-cyclodextrin, β-cyclodextrin, and γ-cyclodextrin	Significant impact on SCFA composition (β-cyclodextrin—highest total SCFA and acetate production; γ-cyclodextrin—highest SCFA and acetate production rates ↑, highest butyrate production; α-cyclodextrin—highest propionate production)	Guevara et al. [60]
In vitro study	Liquid cultures of prebiotic strains	100 μg/mL of α-cyclodextrin, methyl-β-cyclodextrin, and hydroxypropyl-γ-cyclodextrin	Positive effect on growth of prebiotic bacteria in cultures compared to control	Gościniak et al. [12]

* Arrows indicate the direction of changes in the bacterial abundance: ↑ represents an increase, while ↓ represents a decrease.

**Table 3:** Summary of studies on the prebiotic activity of dextrans.

Model	Sample	Findings	Authors
In vitro study	1.0%, *w*/*v* of dextran produced by dextransucrase from *Weissella cibaria* JAG8	Dextran supported the growth of probiotic bacteria (*Bifidobacterium animalis subspecies lactis*, *Bifidobacterium infantis*, and *Lactobacillus acidophilus*) and did not promote the growth of unwanted *E. coli*	Tingirikari et al. [85]
In vitro study	1.0%, *w*/*v* of dextran produced *Weissella cibaria* RBA12	Enhanced growth of probiotic *Bifidobacterium* and *Lactobacillus* spp., and controlled growth of non-probiotic enteric bacteria	Lv et al. [86]
In vitro study	Dextran NEXTDEXT^®^ (2.3 × 10^5^ < MW < 1 × 10^7^ kDa)	Enhanced the relative amount of *Prevotella* and *Bacteroides*, consistently with a favorable acetate–propionate	Amaretti et al. [87]
In vitro study	10 g/L of dextran and novel oligodextrans (I, II, and III) produced in the University of Reading (UK)	Enrichment of *Bifidobacteria* in the batch cultures, with high levels of persistence up to 48 h for dextran and oligodextrans	Olano-Martin et al. [88]
In vitro study	10% *w*/*v*; 0.5 kDa dextran with 25% α-1,2 branching, 1 kDa dextran with 32% α-1,2 branching and inulin	0.5 and 1.0 kDa dextran induce similar effects towards the gut microbiota, but the 0.5 kDa dextran was fermented faster	Sarbini et al. [76]
In vitro study	1.0%, *w*/*v* of systems containing dextran 5 kDa, 40 kD, and 70 kD	Dextran 40 kD proved to be the most effective prebiotic for *Bifidobacterium longum*, *Bifidobacterium animalis*, *Faecalibacterium prausnitzii*, *Lactobacillus salivarius*, *Lactiplantibacillus plantarum 299v*, and *Lacticaseibacillus rhamnosus* GG. Dextran 5 kD exhibited moderate effectiveness for *Lactobacillus helveticus*, while Dextran 70 kD showed the lowest impact	Sip et al. [90]

**Table 4:** Safety concerns about cyclodextrin and dextran.

Feature	Cyclodextrins (CDs)	Dextran
Safety with Oral Use	Generally safe, may cause gastrointestinal problems	Generally safe, but not commonly used orally
Parenteral Use Risks	Nephrotoxicity, hemolysis	Anaphylaxis, hypervolemia, bleeding
Allergic Reactions	Rare but possible with prolonged use	Rare with oral use, much lower risk than with intravenous administration
Toxicity Modulation	Lowered by using modified CDs (e.g., HP-β-CD)	No need for modification in the oral context
